# Distributed solar photovoltaic array location and extent dataset for remote sensing object identification

**DOI:** 10.1038/sdata.2016.106

**Published:** 2016-12-06

**Authors:** Kyle Bradbury, Raghav Saboo, Timothy L. Johnson, Jordan M. Malof, Arjun Devarajan, Wuming Zhang, Leslie M. Collins, Richard G. Newell

**Affiliations:** 1 Energy Initiative, Duke University, Durham, North Carolina 27708, USA; 2 Department of Economics, Duke University, Durham, North Carolina 27708, USA; 3 Nicholas School of the Environment, Duke University, Durham, North Carolina 27708, USA; 4 Department of Electrical & Computer Engineering, Duke University, Durham, North Carolina 27708, USA; 5 Department of Computer Science, Duke University, Durham, North Carolina 27708, USA

## Abstract

Earth-observing remote sensing data, including aerial photography and satellite imagery, offer a snapshot of the world from which we can learn about the state of natural resources and the built environment. The components of energy systems that are visible from above can be automatically assessed with these remote sensing data when processed with machine learning methods. Here, we focus on the information gap in distributed solar photovoltaic (PV) arrays, of which there is limited public data on solar PV deployments at small geographic scales. We created a dataset of solar PV arrays to initiate and develop the process of automatically identifying solar PV locations using remote sensing imagery. This dataset contains the geospatial coordinates and border vertices for over 19,000 solar panels across 601 high-resolution images from four cities in California. Dataset applications include training object detection and other machine learning algorithms that use remote sensing imagery, developing specific algorithms for predictive detection of distributed PV systems, estimating installed PV capacity, and analysis of the socioeconomic correlates of PV deployment.

## Background & Summary

For many years, aerial photography was the primary source of commercial high-resolution imagery, including multispectral color orthoimagery (imagery that has been orthorectified so the image lacks spatial distortion). Since 1999, with the launch of the Ikonos satellite, emerging satellites capable of high spatial resolution (≤ 50 cm) such as GeoEye-1 (41 cm), WorldView-2 (46 cm)^1^, and WorldView-3 (31 cm), produce high-resolution panchromatic imagery with average revisit times of 3 days or less. With both high spatial and temporal resolution, vast quantities of information are available for monitoring and assessment of our environment and resources in near real-time. Automatic object detection methods provide the basis for such assessment.

Machine learning techniques, specifically scene categorization and object detection, provide a means to automate the generation of insight from high-resolution orthoimagery. In scene categorization^2–4^ a semantic label is assigned to an image (or scene) as a whole. In object detection^5–8^, the goal is to identify all instances in the imagery of a particular object type^9–12^ such as roads^13–18^, buildings^19–25^, vehicles^26,27^ solar PV arrays^28^, etc.

The development of supervised object detection techniques requires training data with labelled classes of objects in order to quantitatively measure performance. Several such datasets are publically available for object detection. One limitation of existing publicly available datasets is that they include too few annotated objects for the application of modern classification techniques, such as convolutional neural networks, which require thousands, or even millions of observations^29^. The SZTAKI-INRIA benchmark dataset, for instance, contains 665 labelled buildings in 9 images^30^. Other datasets have limited geographic coverage, including the Vaihingen dataset^31,32^ which provides labelled buildings, roads, trees, cars, vegetation, and artificial groundcover for three regions of the city of Vaihingen, Germany. Still other datasets contain cropped images of many object examples, but do not include precise bounding polygons^33,34^. In this effort, we developed a dataset with nearly 20,000 solar array annotations from multiple cities and diverse settings including urban, suburban, and rural landscapes, with each array identified with a bounding polygon.

Beyond the development of improved object detection algorithms more generally, changes in the energy system have given rise to the need for related data analytic capabilities. The penetration of renewable energy systems, for instance, has been increasing rapidly over the past decade, with solar PV arrays constituting a significant portion of that growth. For grid system operators and decision makers, detailed building-level or neighbourhood-level information on the power capacity and locations of these arrays can enable system operators to plan distribution line topologies to ensure electricity reliability with increased two-way flows of energy. Additionally, building-level or neighborhood-level information on solar PV enables socioeconomic analyses of rooftop PV deployment and the development of predictive algorithms for anticipating future PV array locations.

Presently, there is no central database of individual solar PV array locations and power capacity in the United States. There are national- and state-aggregated databases such as those from the U.S. Energy Information Administration, state-specific databases such as the California Solar Initiative (CSI) dataset^35^ and the North Carolina Utilities Commission^36^, and county-specific collections such as those for the City and County of Honolulu^37^. Compiling this information currently involves extracting building permit data county-by-county, scraping public utility commission databases for interconnection documents, or working with utilities to gain access to the proprietary data under data use agreements.

To provide a publicly available means of generating this granular information for any geographic region of interest, we created a dataset originally collected to train machine learning object detection algorithms to develop the process of automatically identifying solar PV locations using high-resolution orthoimagery. This dataset contains the geospatial coordinates and border vertices for over 19,000 solar panels from four cities in California: Fresno, Stockton, Oxnard, and Modesto. This dataset is useful as both a training dataset for the development of object identification algorithms using remote sensing data more generally, as well as for focused analyses of the deployment patterns of distributed solar PV.

This dataset may also be of interest to researchers working on:

- object identification in remote sensing data,
- machine learning, including techniques requiring large training datasets (such as convolutional neural networks),
- socioeconomic analyses of renewable energy resource development, and
- electric power grid and microgrid analysis for distributed generation.

## Methods

**Figure 1:**
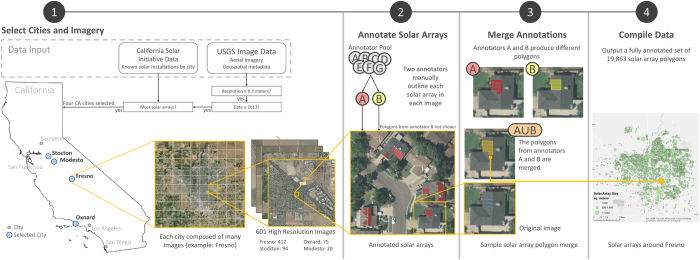
Flowchart showing the dataset generation process. This includes (1) selecting imagery from four cities, (2) manually annotating solar array polygons, (3) merging the polygons identified by multiple annotators, and (4) compiling the resulting dataset.

We constructed this dataset to contain the location and bounding polygon for solar PV arrays within cities having a large number of solar PV arrays as well as the necessary imagery. Our process is illustrated in Fig. 1. Through this process, we (1) carefully selected our imagery data, (2) manually annotated the locations and boundaries of every solar panel in each image using two different annotators, (3) merged the annotations of each user into a single dataset, and (4) compiled the results.

### Select cities and imagery

The United States Geological Survey (USGS) has an extensive collection of publicly available high-resolution aerial orthoimagery from across the United States^38^. Our criteria for selecting a city were twofold: (a) the resolution of the imagery should be high—no lower than 30cm, and (b) the city should have many solar arrays. Since solar panels are typically between 1 and 2 meters in length, we would want every panel to be represented by multiple pixels in the image, and a 30 cm resolution threshold enables that. Ensuring there are many solar panels in a city was a more challenging task. To determine how many solar arrays are in a given city, we referred to the California Solar Initiative dataset, which provides listings of solar arrays by city. The vast majority of those solar arrays were installed after 2012, so we limited our selection to images that had been taken in 2013 or later.

Using these two criteria, we identified the California cities with USGS orthoimagery data^38^ of 30 cm or finer, taken during or after 2013, and we sorted them by the number of solar arrays in each city. This process yielded four cities with 30cm resolution: Fresno (including the neighboring city of Clovis), Stockton, Modesto, and Oxnard. For Fresno, Oxnard, and Stockton, we included the entire city, but for Modesto we chose a representative subset of images from the city, since the solar panels were less densely deployed in this city. We then acquired 601 TIF images^38^ from these cities to annotate: 412 from Fresno, 94 from Stockton, 75 from Oxnard, and 20 from Modesto. Each image is 5000-by-5000 pixels and represents an area of 2.25 square kilometers (0.87 square miles, or 556 acres).

### Annotate solar arrays

With the USGS data for the four cities, we manually annotated the polygonal boundaries of every solar array. Using a MATLAB-based graphical user interface (GUI) developed for this purpose, a team of researchers divided the imagery data and manually drew a polygon around each solar panel seen in the imagery. As a researcher annotated each image, the GUI would present a subset of the image file, moving left to right, top to bottom throughout the image to ensure the annotator visually inspected the entirety of every image. The GUI allowed users to save or delete the polygons they had created and to display existing polygons that they had previously annotated, to ensure they didn’t duplicate their annotations.

Once the annotator completed the image, the vertices of each polygon that had been drawn were saved as pixel coordinates within that image. From those vertices we computed the centroid of each polygon and saved those along with the pixel coordinates. The pixel coordinates were then converted into longitude and latitude coordinates through two steps. First the Universal Transverse Mercator (UTM) coordinates were estimated as linear interpolations of the pixel distance from the northwest and southeast vertices of the USGS orthoimage containing the solar array. The UTM coordinates were then converted to latitude and longitude coordinates using the datum for that image file.

Manual solar panel annotation on the scale of this dataset (over 19,000 distinct objects) required steps to ensure quality and to prevent incorrect labelling or omission of solar arrays. To ensure each solar array was accurately identified in the data, two annotators processed each image file independently.

### Merge annotations

The results from each annotation were compared with one another and merged, with a confidence value provided to account for the level of agreement between the two annotators. After two annotators identified solar array polygons in each image, we merged (via a union) those polygons that overlap, producing a single, non-redundant set of solar array polygons. The confidence value that we associate with each merged polygon was calculated by applying the Jaccard Similarity Index^39^ which, in our case, is the intersecting area between two polygons divided by the unioned area between those polygons. Assuming the area of one polygon is *A* and a second polygon is *B*, the Jaccard index, *J*(*A*, *B*), describing the similarity between those two polygons is given by Equation (1).

(1)J(A,B)=|A∩B||A∪B|

The Jaccard index is bounded such that 0≤*J*(*A*, *B*)≤1, which makes interpreting the results clear. A value of zero indicates the two polygons are disjoint, without overlap, while a Jaccard index of one indicates that *A* and *B* are identical, completely overlapping polygons.

For a small number of polygons in the dataset, there is overlap between more than two polygons. For these rare cases, all overlapping polygons were unioned and the reported confidence value was given as the sum of the pairwise intersections divided by the total unioned area. In this way, we defined the confidence value for *N* polygonal areas as given in Equation (2).

(2)(A1,A2,…,AN)=∑i≠j|Ai∩Aj||Ui=1NAi|

We restricted the index of Equation (2) to a maximum value of 1. This allows for every solar array that was identified to have an associated confidence value based on the degree of agreement between annotators.

### Compile data

**Figure 2:**
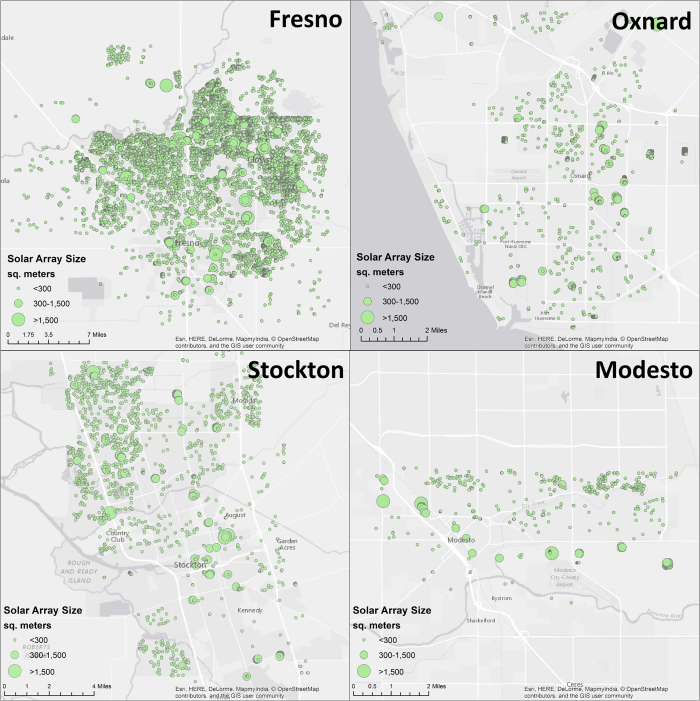
Location and area (m^2^) of the manually annotated solar arrays for the Fresno, Oxnard, Stockton, and Modesto regions.

The compiled data for all annotated polygons along with other identifying metadata linking the polygon to the image file and longitude and latitude coordinates were placed in a MATLAB cell array and uploaded to the Figshare data repository (Data Citation 1). The resulting collection of data is shown across the four cities in Fig. 2, where both the location and size of the solar arrays (in square meters) can be seen.

## Data Records

The solar array polygon location and extent data as well as the imagery data used to generate this dataset are available as a collected repository of four sets of files (Data Citation 1). The primary dataset within that collection (Data Citation 2) consists of the table of solar panels in four formats with equivalent information to enable these data to be as easily accessed as possible: JSON, GeoJSON, MATLAB (.mat) and comma-separated values (CSV) files. A complete listing of each field, its contents, the format of the data, and the units of the data is given in Table 1.

The first of the files is in JavaScript Object Notation (JSON) data-interchange format. The JSON file is titled *SolarArrayPolygons.json* and each object in this array has fields that correspond to those described in Table 1. The second of the files is a GeoJSON file, *SolarArrayPolygons.geojson,* which has the latitude and longitude coordinates of each array stored along with all of the properties of each array (described in Table 1), including the pixel-based coordinates.

For the MATLAB format, a single file titled *SolarArrayPolygons.mat* contains ‘data’, the cell array containing all of the columns described in Table 1, as well as an array called ‘fields’ which includes the name of each column of the cell array ‘data’.

These data are also presented in an alternative, and perhaps more broadly accessible CSV format. There are three CSV files. The file *polygonDataExceptVertices.csv* contains the 19 fields of Table 1, which is all of the data except for the vertices of the bounding polygons for each array. The vertices are presented in two files, both containing the same polygons: one file contains the coordinates of the vertices as pixel (x,y) values with respect to the image that contains the polygon (*polygonVertices_PixelCoordinates.csv*) while the other file contains latitude and longitude coordinates for each vertex (*polygonVertices_LatitudeLongitude.csv*). These two CSV files with polygon vertices also contain a column (the second column of the files) with the number of vertices in the polygon, to make reading the data from the CSV files more straightforward. The polygon ID field, which is the first column in all of these files, links the information between each of the CSV files.

All imagery data is from the USGS High Resolution Orthoimagery collection and can be found in Data Citations 3–6. For Fresno (Data Citation 3), Stockton (Data Citation 4), and Modesto (Data Citation 5), each image file actually has two files associated with it: *<IMAGENAME>.tif*, which is the image file itself, and *<IMAGENAME>.tif.xml*, which is the USGS metadata for the corresponding image. The image names correspond to the ‘image_name’ field of Table 1 found in the files described earlier (Data Citation 2). The Oxnard imagery (Data Citation 6) also has a set of image files *<IMAGENAME>.tif*, but only one metadata file for the entire imagery set, which is *cirgis_2013_12inch_metadata.xml*.

## Technical Validation

The task of manually annotating objects in remote sensing imagery to produce ground truthed data is tedious, requiring many person-hours to complete. There are three potential sources of error in this dataset that we controlled for: (1) missed solar arrays, (2) regions that are not solar arrays, but are labelled incorrectly as solar arrays, (3) inaccurately drawn polygons around solar arrays. We discuss each of these sources of error, and describe our approach to minimizing the effect of that error.

1. *Missed solar arrays*. Relying on human annotators requires that the annotator actually see the solar array, and it is not always the case that every annotator will see every array. We systematized this process through our annotation GUI to ensure that the eyes of each annotator passed over every (collectively exhaustive) subimage of each image to maximize the probability of visual detection for each solar array. Additionally, two annotators processed every image we annotated to further reduce the probability of missing a solar array.
2. *Incorrectly identified solar arrays*. Solar panels, although typically quite visually distinct, may occasionally be confused for other objects, including solar thermal collectors, skylights, or various pieces of rooftop equipment. Sun glare and time of day can also change the appearance of objects in satellite imagery. We provided training for annotators to make them aware of these pitfalls so that they were informed as to what a solar array typically looks like and to ensure consistency between annotators.
3. *Incorrectly drawn polygons*. The accuracy of the polygons drawn around each solar array is subject to the resolution of the image and the precision of the annotator. As an assay, we computed the Jaccard index as part of the annotated polygon merging process to provide a measure of agreement between each annotation. These values were analysed below and suggest that typically when two annotators both identify a solar array, their annotations strongly agree. In all cases, but particularly relevant when there is disagreement between annotators, we provide the Jaccard index for each final annotation so that users may filter the data based on the needs of their application.

### Analysis of confidence values

**Figure 3:**
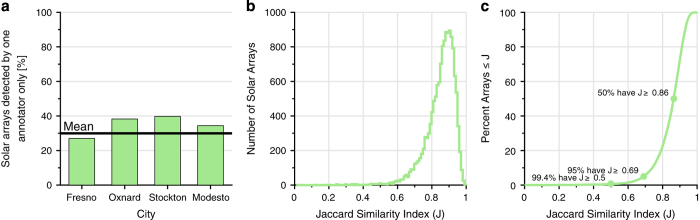
Analysis of manual solar array polygon annotations. (**a**) Shows the percent of identified solar arrays in each city identified by only one annotator (on average this was 30%, so 70% were identified by two annotators). Of those solar arrays identified by more than one annotator (**b**) shows the histogram of Jaccard Indices and (**c**) shows the cumulative percent of annotated arrays with a Jaccard index less than or equal to a given value, J. 99.4% have a Jaccard Index greater than 0.5, and 95% have a Jaccard Index greater than 0.69.

The Jaccard Similarity Indices provide an indication of how similar any two polygonal annotations are, and whether or not only one or both of the annotators identified a region as a solar array. Across the 19,863 polygons in the dataset, Fig. 3a shows that on average, across all the images, about 30% of polygons were identified by only one annotator; therefore, 70% were identified by two annotators. The annotators were visually scanning hundreds of square miles of land for relatively small features within the image, therefore it is not surprising for an individual annotator to miss some number of solar arrays. The distribution of Jaccard Index values shown in Fig. 3b demonstrates that for those polygons identified by more than one annotator, the Jaccard Index was high on average, and Fig. 3c shows that 99.4% have a Jaccard Index greater than 0.5, 95% have a Jaccard Index greater than 0.69, and 50% have an Index greater than 0.86. For reference, assuming both polygons consisted of about 24 pixels in an image, a Jaccard index of 0.86 is a difference of about 4 pixels.

## Usage Notes

The ground truth data files are provided in .json, .geojson, .csv and .mat formats. For most users the .json or .geojson files will likely be the preferred format. The comma separated value (CSV) format should also be readily accessible and the *polygonDataExceptVertices.csv* file contains information that can be used easily to get locations and sizes of all of the solar arrays in the dataset. Extracting the polygon vertices from either *polygonVertices_PixelCoordinates.csv* or *polygonVertices_LatitudeLongitude.csv* can be accomplished by reading in each row of data, knowing the first two columns are the ID and the number of vertices, then using the number of vertices to extract exactly that many pairs of coordinates from that row. The .mat file may be opened by either MATLAB or Octave, and is in a cell array format to make extracting the polygon vertices of each solar array boundary easier to access and use. The imagery files from the USGS are TIF files that are readable in most computational environments.

This dataset contains both geographic coordinates (longitude and latitude) as well as pixel coordinates. In processing the data, the use of pixels coordinates removes the need for a full GIS environment, for those who prefer to conduct an image processing or machine learning analysis without projecting the data into a specific coordinate system.

Additionally, since each solar array entry in the dataset contains a reference to the image file that contains it, a user may choose to select a smaller subset of images, or even just a single image and the dataset can be filtered to only those solar array polygons occurring in that image.

## Additional Information

**How to this article**: Bradbury, K. *et al.* Distributed solar photovoltaic array location and extent dataset for remote sensing object identification. *Sci. Data* 3:160106 doi: 10.1038/sdata.2016.106 (2016).

**Publisher’s note**: Springer Nature remains neutral with regard to jurisdictional claims in published maps and institutional affiliations.

## Supplementary Material



## Figures and Tables

**Table 1:** Data field descriptions.

**Field**	**Field**/**Column Name**	**Description**	**Format**	**Units**
Polygon ID	polygon_id	Unique identifier for each polygon	Integer	N/A
Centroid Latitude	centroid_latitude	Latitude of the centroid of the polygon bordering the solar array given	Double	Decimal Latitude
Centroid Longitude	centroid_longitude	Longitude of the centroid of the polygon bordering the solar array given	Double	Decimal Longitude
Centroid Pixel y-coordinate	centroid_latitude_pixels	Pixel y-coordinate of the centroid of the polygon bordering the solar array with respect the image file containing the solar array (origin is 0,0)	Double	Pixels
Centroid Pixel x-coordinate	centroid_longitude_pixels	Pixel x-coordinate of the centroid of the polygon bordering the solar array with respect the image file containing the solar array (origin is 0,0)	Double	Pixels
City	city	Name of the city the solar array is located in	String	N/A
Area of Polygon (pixels)	area_pixels	Area of the solar array in square pixels	Double	Pixels^2^
Area of Polygon (meters)	area_meters	Area of the solar array in square meters	Double	Meters^2^
Image Name	image_name	Name of the image file containing this solar array	String	N/A
Latitude Northwest Corner of Image Coordinates	nw_corner_of_image_latitude	Latitude of the northwest corner of Image containing this solar array	Double	Decimal Latitude
Longitude Northwest Corner of Image Coordinates	nw_corner_of_image_longitude	Longitude of the northwest corner of Image containing this solar array	Double	Decimal Longitude
Latitude Southeast Corner of Image Coordinates	se_corner_of_image_latitude	Latitude of the southeast corner of Image containing this solar array	Double	Decimal Latitude
Longitude Southeast Corner of Image Coordinates	se_corner_of_image_longitude	Longitude of the southeast corner of Image containing this solar array	Double	Decimal Longitude
Datum	datum	Datum of Image	String	N/A
Projection	projection_zone	Projection Zone of Image	String	N/A
Resolution	resolution	Resolution of Image	Integer	Meters^2^/Pixel
Jaccard Index	jaccard_index	Jaccard Similarity Index of the merged polygons	Double	N/A
Polygon Vertices (Pixels y,x)	polygon_vertices_lat_lon	Array Vertices of the Polygon in Pixels	Array of [2×1] vectors of doubles	Latitude, Longitude
Polygon Vertices (Lat,Lon)	polygon_vertices_pixels	Array Vertices of the Polygon in Latitude, Longitude	Array of [2×1] vectors of doubles	Pixels
